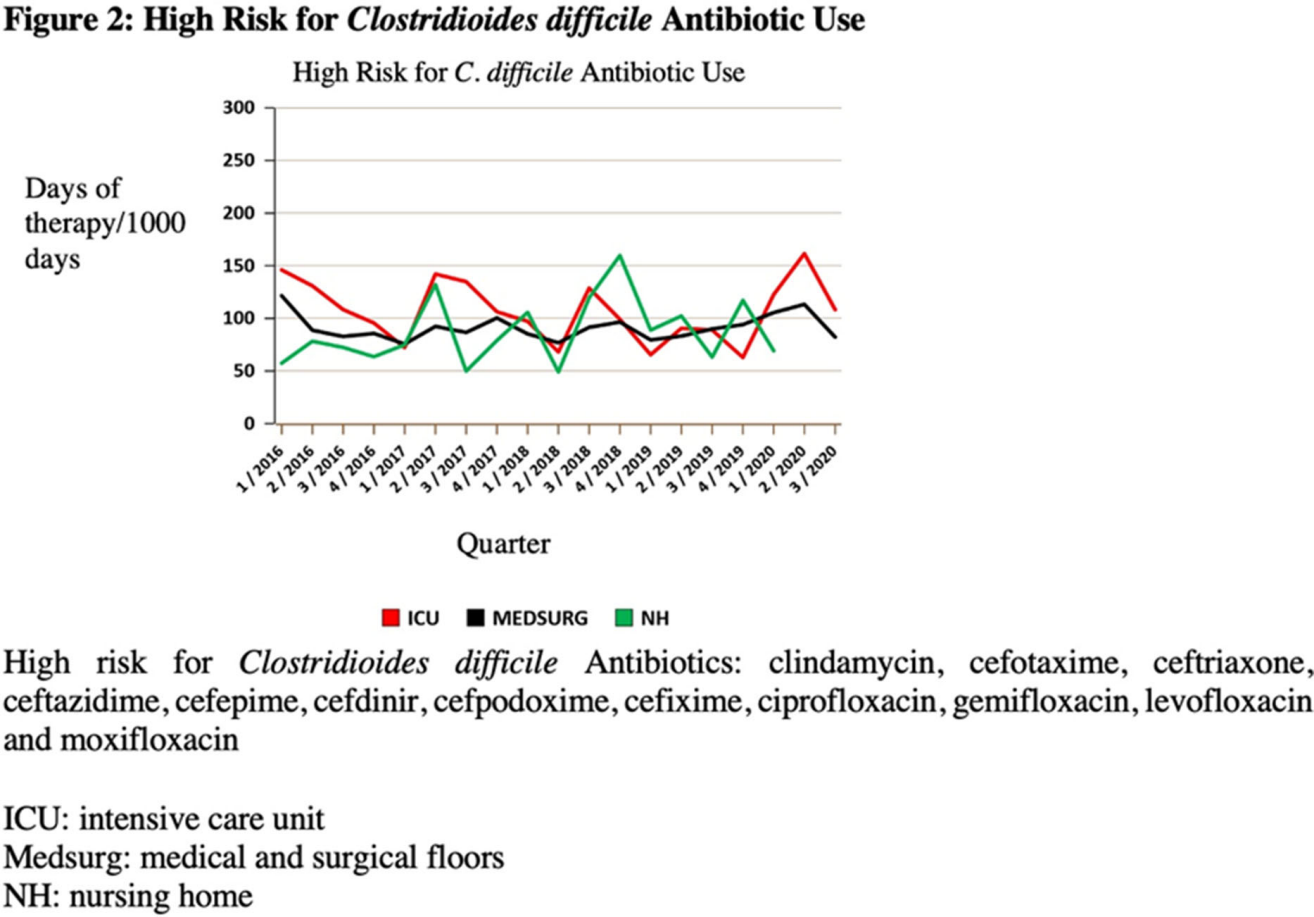# Did *Clostridioides difficile* Testing and Infection Rates Change During the COVID-19 Pandemic?

**DOI:** 10.1017/ash.2021.79

**Published:** 2021-07-29

**Authors:** Armani Hawes, Payal Patel, Angel Desai

## Abstract

**Background:** The COVID-19 pandemic has underscored the importance of ongoing infection prevention efforts. Increased adherence to infection prevention recommendations, increased antibiotic use, improved hand hygiene, and correct donning and doffing of personal protective equipment may have influenced healthcare-associated infections (HAIs) in the United States during the pandemic. In this study, we investigated testing for *Clostridioides difficile* infection (CDI) and incidence during the initial surge of the pandemic. We hypothesized that strict adherence to contact precautions may have resulted in a decreased incidence of CDI in hospitalized patients during the first peak of the COVID-19 pandemic and that CDI testing may have increased even in the absence of directed diagnostic stewardship efforts. **Methods:** We conducted a single-center, retrospective, observational study at the Veterans’ Affairs (VA) Hospital in Ann Arbor, Michigan, between January 2019 and June 2020. We compared data on CDI tests from January 2019 through February 2020 to data from March 2020 (the admission of the first patient with COVID-19 at our institution) through June 2020. Pre-peak and peak periods were defined by confirmed cases in Washtenaw County. No novel diagnostic or CDI-focused stewardship interventions were introduced by the antimicrobial stewardship program during the study period. An interrupted time series analysis was performed using STATA version 16.1 software (StataCorp LLC, College Station, TX). **Results:** There were 6,525 admissions and 34,533 bed days between January 1, 2019, and June 30, 2020. Also, 900 enzyme immunoassay (EIA) tests were obtained and 104 positive cases of CDI were detected between January 2019 and June 2020. A statistically significant decrease in EIA tests occurred after March 1, 2020 (the COVID-19 peak in our region) compared to January 1, 2019–March 1, 2020 (Figure 1). After March 1, 2020, the number of EIA tests obtained decreased by 10.2 each month (95% CI, −18.7 to −1.7; *P* = .02). No statistically significant change in the incidence of CDI occurred. The use of antibiotics that were defined as high risk for CDI increased in the months of April–June 2020 (Figure 2). **Conclusions:** In this single-center study, we observed a stable incidence of CDI but decreased testing during the first peak of the COVID-19 pandemic. Understanding local HAI reporting is critical because changes in HAI reporting structures and exemptions during this period may have affected national reporting. Further research should be undertaken to investigate the effect of COVID-19 on other HAI reporting within the US healthcare system.

**Funding:** No

**Disclosures:** None

**Figure 1.**
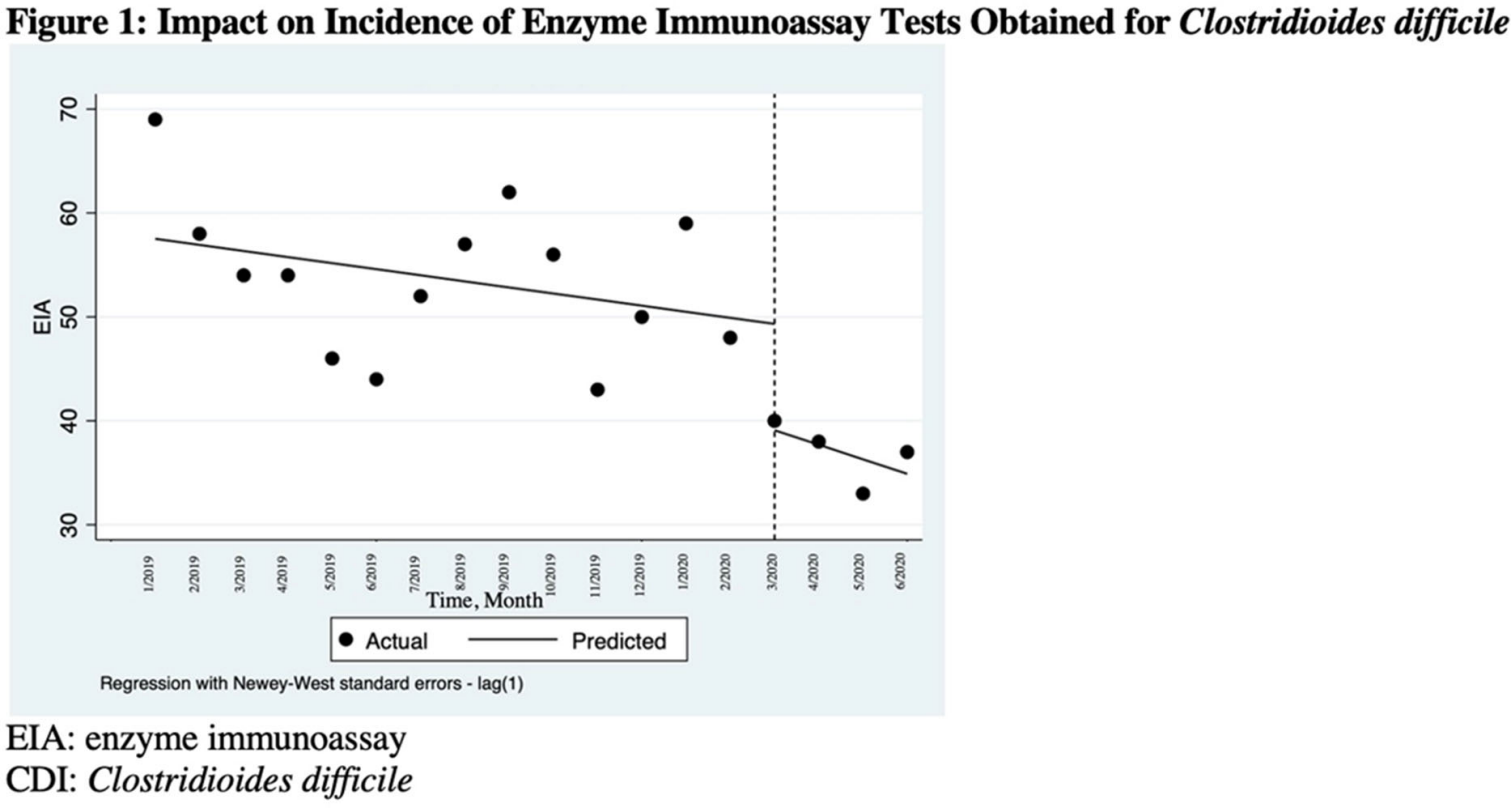

**Figure 2.**